# COVID-19 AND IBD: LOOK BEYOND THE VIRUS. A SYSTEMATIC REVIEW AND META-ANALYSIS FOCUSED ON BIOLOGIC THERAPY CONTINUITY

**DOI:** 10.1590/S0004-2803.24612025-147

**Published:** 2026-09-21

**Authors:** Mariana ROLIM, Andrea Benevides LEITE, Lauro Vieira PERDIGÃO, Anna Sara LEVIN

**Affiliations:** 1 Universidade de São Paulo, Faculdade de Medicina, Departamento de Doenças Infecciosas, São Paulo, SP, Brasil.; 2 Universidade Christus (Unichristus), Faculdade de Medicina, Fortaleza, Ceará, Brasil.; 3 Universidade de Fortaleza (UNIFOR), Faculdade de Medicina, Centro de Ciências da Saúde, Fortaleza, Ceará, Brasil.; 4 Hospital Geral de Fortaleza, Fortaleza, CE, Brasil.

**Keywords:** Inflammatory bowel diseases, COVID-19, SARS-CoV-2, biological, treatment adherence and compliance, Doenças inflamatórias intestinais, COVID-19, SARS-CoV-2, biológicos, adesão à medicação

## Abstract

**Background/Objective:**

Whether COVID-19 worsens the longterm course of inflammatory bowel disease (IBD) remains uncertain. We synthesized comparative cohorts to assess IBD outcomes after SARS-CoV-2 infection versus noninfected IBD controls and to evaluate the impact of biologic discontinuation.

**Methods:**

Systematic review (PRISMA 2020). Adult IBD cohorts with vs without prior SARS-CoV-2. Databases: MEDLINE (PubMed), Web of Science, and Scopus; reference lists screened. Time window: Jan 2020 to Dec 2024. Risk of bias: Newcastle-Ottawa. Randomeffects Mantel-Haenszel with REML variance and Hartung-Knapp adjustment; heterogeneity by I^2^/τ^2^/Q. Zero events handled via 0.5 continuity correction; sensitivity analyses reported risk differences (percentage points) and studylevel effects for sparse outcomes. Certainty graded with GRADE.

**Results:**

Four matched cohorts (n≈1.3k; Italy/USA). Prior SARS-CoV-2 did not worsen IBD clinical course (OR: 1.06; 95%CI: 0.76-1.49), nor alter UC extent (OR: 1.26; 95%CI: 0.33-4.83) or CD location/behavior (OR: 1.00; 95%CI: 0.06-16.15 / OR: 1.00; 95%CI: 0.46-2.18). Conversely, COVID-19 associated with biologic delay/discontinuation (OR: 10.44; 95%CI: 3.56-30.62). Across studies, flares were more frequent when biologics were delayed/stopped (~52% vs ~18%; RD ≈ +34 p.p.).

**Conclusion:**

COVID-19 per se does not appear to aggravate IBD outcomes; the clinically actionable signal is treatment continuity. In IBD patients without highrisk features for severe COVID-19, maintaining biologics should be considered to prevent flares.

## HIGHLIGHTS

• SARS-CoV-2 infection did not worsen inflammatory bowel disease (IBD) outcomes.

• Stopping or delaying biologics during COVID-19 was linked to much higher flare rates (≈52% vs 18%).

• Continuing biologics in patients without high-risk features for severe COVID-19 should be considered, given the higher flare rates observed after treatment discontinuation

• Look beyond the virus, also keep IBD control in focus: anticipate flares and avoid reflexive treatment pauses in low-risk COVID cases.

## RESUMO

**Contexto/Objetivo:**

Ainda é incerto se a COVID-19 piora o curso clínico da doença inflamatória intestinal (DII). Sintetizamos coortes comparativas para avaliar desfechos da DII após infecção por SARS-CoV-2 versus controles com DII não infectados e estimar o impacto da descontinuação de biológicos.

**Métodos:**

Revisão sistemática (PRISMA 2020). Coortes adultas com DII com vs sem infecção prévia por SARS-CoV-2. Bases: MEDLINE (PubMed), Web of Science e Scopus; listas de referências examinadas. Período: jan/2020 a dez/2024. Risco de viés: Newcastle-Ottawa. Metanálise de efeito aleatório (Mantel-Haenszel) com variância REML e ajuste Hartung-Knapp; heterogeneidade por I^2^/τ^2^/Q. Eventos zero tratados com correção 0,5; análises de sensibilidade com diferença absoluta de risco (pontos percentuais) e efeitos por estudo em desfechos esparsos. Certeza avaliada por GRADE.

**Resultados:**

Quatro coortes pareadas (n≈1,3 mil; Itália/EUA). A infecção prévia por SARS-CoV-2 não piorou o curso clínico da DII (OR: 1.06; 95%CI: 0.76-1.49), nem alterou a extensão da RCU (OR: 1.26; 95%CI: 0.33-4.83) ou localização/comportamento do DC (OR: 1.00; 95%CI: 0.06-16.15 / OR: 1.00; 95%CI: 0.46-2.18). Por outro lado, a COVID-19 associou-se ao atraso/descontinuação de biológicos (OR: 10.44; 95%CI: 3.56-30.62). Entre os estudos, exacerbações foram mais frequentes quando biológicos foram adiados/suspensos (~52% vs ~18%; +34 p.p.).

**Conclusão:**

A COVID-19 per se não parece agravar desfechos da DII; porém a descontinuação do tratamento é um fator decisivo para a ocorrência de flares da doença. Em pacientes sem alto risco para COVID-19 grave, a manutenção de biológicos deve ser considerada para prevenir complicações da DII.

## INTRODUCTION

The COVID-19 pandemic was particularly fearful for patients with inflammatory bowel disease (IBD), which refers to Crohn’s disease (CD) and ulcerative colitis (UC). IBD is a multi-faceted illness, predisposing patients towards infection and malnutrition, and is associated with a high burden of hospitalization, surgery, and frequent use of immunosuppressive therapy^1^.

Multiple studies have been published on the impact of IBD on COVID-19 outcomes. Data suggest that IBD individuals do not seem to be at a higher risk of being infected by SARS-CoV-2 than the general population^2^, but the use of salicylates seems to increase this risk^3^. IBD is not associated with increased COVID-19 hospitalization, intensive care unit admission, or death^4^, but multiple studies identified IBD activity to be an independent risk factor for developing severe COVID-19^5^ ^,^ ^6^, mainly those with IBD under the age of 50 who are actively flaring are at higher risk of severe COVID-19 outcomes^2^. The use of immunosuppressants or immunobiological drugs did not change the clinical presentation of COVID-19^3^ and was not associated with an increased risk of more severe COVID-19 outcomes^7^ ^,^ ^8^.

Therefore, over the last four years, since the onset of the pandemic, the knowledge of the impact of IBD on COVID-19 has expanded dramatically. However, little is known about the course of IBD after COVID-19. Most of the current evidence has not been evaluated in systematic reviews or is outdated. In this systematic review and meta-analysis^9^, we aim to evaluate the impact of SARS-CoV-2 infection on the clinical course and treatment of IBD.

## METHODS

This systematic review was registered in the PROSPERO database (registration number: CRD420261366700). It was conducted in accordance with the Preferred Reporting Items for Systematic Reviews and Meta-Analyses (PRISMA) statement.

### Search strategy

Bibliographical research for published observational studies was carried out in three databases - PubMed, Web of Science, and Scopus from 2020 until December 2024. The following search terms were combined: “COVID-19”, “SARS-CoV-2”, “Inflammatory Bowel Diseases”, “Patient Outcome Assessment”, “Health Care”, “Therapeutics”, “Drug Therapy”, “Treatment Outcome”, “Treatment Switching”, “Complications”, “Surgery”. Previous reviews and meta-analyses, as well as the reference lists of the selected studies, were also screened to complete the literature search.

### Study selection

Two researchers independently screened all relevant titles and abstracts of retrieved publications to identify eligible studies, and then reviewed the full texts of the selected articles. Inclusion criteria were applied to selected full texts to identify potentially eligible articles. Inconsistencies in data collection were resolved by a third researcher. The identification, screening, and inclusion process/excluding articles were illustrated using a PRISMA flowchart.

### Inclusion criteria

Observational studies performed on humans with IBD aged 18 years or older who had a SARS-CoV-2 infection analyzed the outcomes of IBD after COVID-19 infection and had a comparison group composed of IBD patients without SARS-CoV-2 infection. All included studies required laboratory confirmation of SARS-CoV-2 infection (PCR, antigen, or serology), and no study included patients based solely on clinical symptoms.

### Data extraction

Data extraction was performed in duplicate, independently, and any differences in data extraction were discussed between authors and resolved. Data were extracted included the following fields: country of study and year of publication, number of patients and type of IBD, age, gender, use of biological therapy (infliximab, adalimumab, certolizumab, vedolizumab, or ustekinumab), duration of follow-up, diagnosis of COVID-19 or SARS-CoV-2 infection, and the outcomes: (1) clinical worsening of IBD, (2) change of IBD therapy, (3) delay or discontinuation of biological therapy, (4) increase in fecal calprotectin, (5) progression of UC extent, (6) change in CD location, and (7) change in CD behavior (non-structuring and non-penetrating, structuring, penetrating). Worsening of IBD was defined as clinical relapse, with Mayo partial score^10^ ≥3 for UC; Harvey-Bradshaw Index^11^ ≥5 for CD; the need for therapeutic intensification, with corticosteroids, biological therapy, or immunosuppressors; hospitalization due to IBD activity; need of surgery for IBD; or transition from remission to any degree of active disease. Therapy delay was defined as a postponement of treatment by at least 14 days. COVID-19 was classified as severe if a symptomatic patient required hospitalization, including stays in the ICU or oxygen supplementation.

### Risk of bias in included studies

The quality of the studies included in this systematic review was scored by two researchers independently using the Newcastle-Ottawa Quality Assessment Scale^12^. Discrepancies were solved by consensus. This tool assesses the risk of bias according to eight items, categorized into three domains (selection, comparability, and exposure or outcome, for case-control and cohort studies, respectively). Each favorable attribute earns 1 to 2 points, and the score can vary from a minimum of 0 points to a maximum of 9 points. A good quality score requires three or four stars in the selection dimension, one or two in the comparability dimension, and two or three stars in the exposure/outcome dimension (exposure /outcomes)^13^.

### Analysis of the data obtained

A narrative synthesis was performed for the studies selected for this review. The data were analyzed to compare results between IBD-infected and non-infected groups. The odds ratio was calculated after grouping the results by outcome. We performed random-effects Mantel-Haenszel meta-analyses using REML variance and Hartung-Knapp adjustment. Heterogeneity was summarized with I^2^, τ^2^, and Cochran’s Q. For sparse/zero-event outcomes, we applied a 0.5 continuity correction; when pooled estimates were unstable, we reported study-level effects and risk differences as sensitivity analyses. Small-study effects were not assessed given <10 studies per outcome. Statistical analyses were performed using the Review Manager (RevMan) program, version 5.3 (Copenhagen: The Nordic Cochrane Center, The Cochrane Collaboration, 2014), and the SPSS for Windows v. 23, IBM Inc. Forest plots were constructed to represent the heterogeneity of the studies.

## RESULTS

**FIGURE 1:**
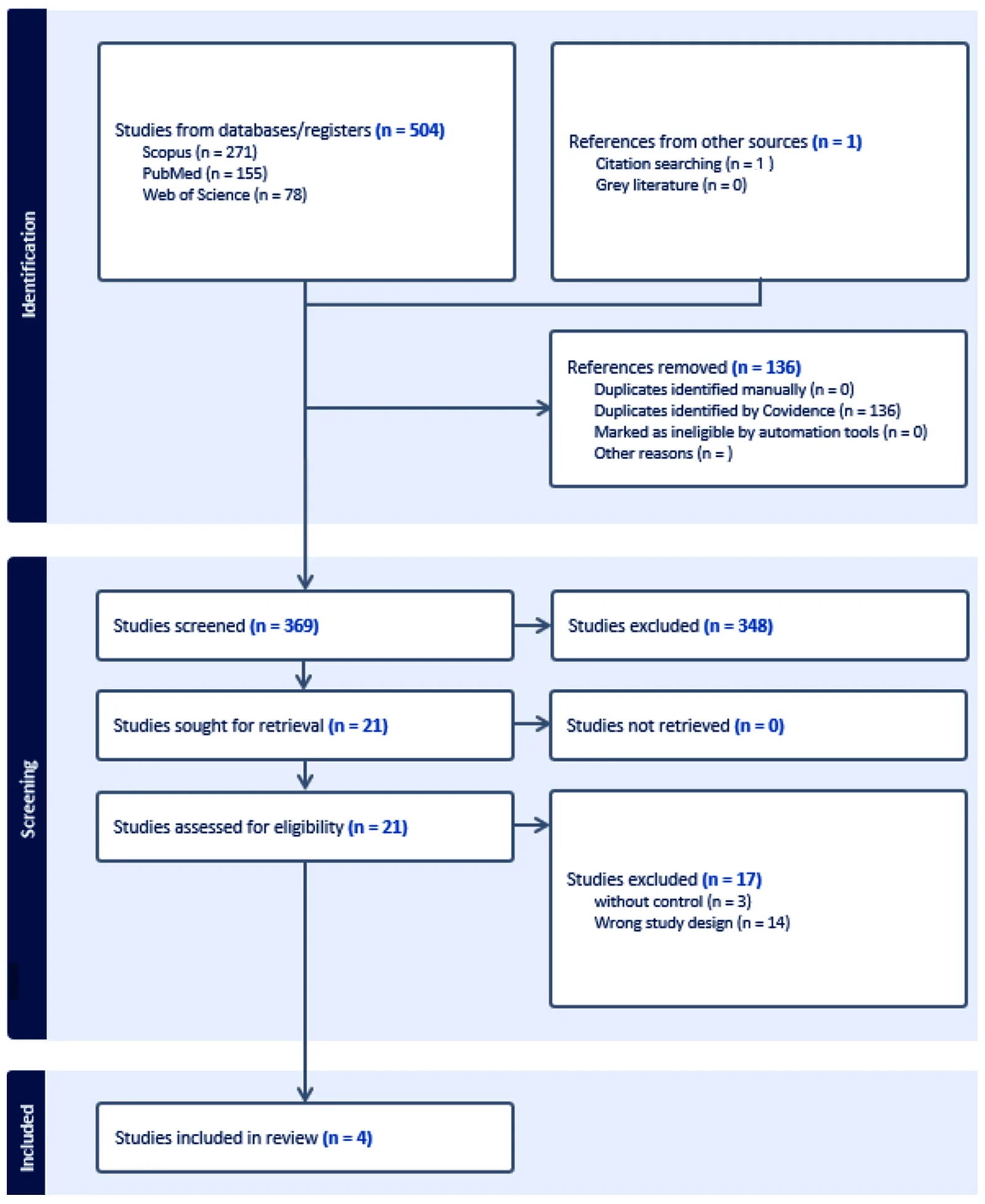
Flow diagram of published studies included in the review (time window: Jan 2020 to Dec 2024).

The initial search yielded 505 studies; after removing duplicates, 369 remained. According to the established criteria, 365 results were excluded. Four studies met the aforementioned inclusion criteria and were included in the systematic review (Figure 1).

All studies in this review scored seven or eight using the Newcastle-Ottawa Scale, representing a low risk of bias (Table 1).

**TABLE 1:** Newcastle-Ottawa Quality Assessment Form for matched cohort.

Author	Year	Selection	Compararability	Outcome	Total
Hong S.J. et al.^14^	2022	****	*	***	********
Bezzio C. et al.^15^	2023	****	*	**	*******
Neri B. et al.^16^	2023	****	*	***	********
Papa A et al.^17^	2022	****	*	***	********

The four studies were conducted in Italy and the USA. All studies compared two groups of IBD patients: those with SARS-CoV-2 infection and those who were not infected. The total of IBD patients was 1343, of which 594 patients had SARS-CoV-2 infection, and 749 did not have SARS-CoV-2 infection. During follow-up, 32 patients were lost, 17 of which had a SARS-CoV-2 infection (of these, seven died from COVID-19). Participants’ ages ranged from 18 to 85 years. The number of patients included in each study varied from 118 to 502. The follow-up period ranged from 14 to 83 weeks (Table 2).

**TABLE 2:** Characteristics of the studies of the impact of infection by SARS-CoV-2 or COVID-19 on the course of Inflammatory Bowel Disease.

	Papa A. 2022	Hong S.J. 2022	Bezzio C. 2023	Neri B. 2023
Country of the study	Italy	USA	Italy	Italy
Definition of exposed group	IBD with SARS-CoV-2 positive oropharyngeal molecular swab	IBD with COVID-19 symptoms and a positive naso pharyngeal SARS-CoV-2 PCR test at the time of symptoms and/or subsequent positive SARS CoV-2-IgG antibody test	IBD diagnosis for at least 6 months and a polymerase chain of SARS CoV-2 positive in a nasopharyngeal swab.	IBD with a recent history (<2 months) of well-defined diagnosis of SARS-CoV-2 infection (molecular or antigenic nasopharyngeal swab)
Definition of non-exposed group	IBD without SARS Cov-2 infection. Do not clearly describe the need for a negative test.	IBD without COVID-19 symptoms PLUS one negative SARS-CoV-2 PCR and at least one negative SARS-CoV-2 IgG	IBD without a SARS-CoV-2 infection. Do not clearly describe the need for a negative test	IBD with no history of previous SARS-CoV-2 infection. Do not clearly describe the need for a negative test
Matching of exposed and non-exposed	1:2 based on diagnosis (CD or UC) and the biologic therapy used	1:1 based on sex, age, and diagnosis (CD or UC)	1:1 based on sex, age, diagnosis (CD or UC), therapy, and disease activity	1:4
Total of patients (exposed/ non-exposed)	285 (95/190)	502 (251/251)	438 (219/219)	118 (29/89)
Age (years) (exposed/non-exposed)	40 (30-55) / 42 (32-44)	39 (29-52) / 38 (30-53) (matched)	45 (18-80) / 46 (19-85) (matched)	43 (30-69) / 53 (20-82)
Male sex (exposed/non-exposed)	49 (51.6%) / 109 (57.4%)	114 (45%) / 114 (45%) (matched)	119 (54.3%) / 119 (54.3%) (matched)	16 (55.1%) / 59 (66.2%)
CD diagnosis (exposed/non-exposed)	192 (64, 67.4% / 128, 67.4%) (matched)	278 (139, 55 % / 139, 55%) (matched)	184 (92, 42% / 92, 42%) (matched)	61 (17, 59% / 44, 49.3%)
UC diagnosis (exposed/non-exposed)	93 (31, 32.6% / 62, 32.6%) (matched)	224 (112, 45% / 112 (45%) (matched)	254 (127, 58% / 127, 58%) (matched)	57 (12, 41.3% / 45, 50.5%)
Biologic therapy (exposed/non-exposed)	95 (100%) / 190 (100%) (matched based on biological therapy)	182 (73%) / 160 (64%)	121 (55%) / 119 (54%) (matched)	11 (38%) / 34 (38,2%)
Anti-TNF biologic therapy (exposed/non-exposed)	72 (75%) / 148 (78%) (matched)	155 (62%) / 130 (52%)	76 (35%) / 69 (31%)	8 (27%) / 26 (29%)
Baseline IBD activity (exposed/non-exposed)	0 (All patients were in remission at baseline)	NA (IBD worsening was defined as hospitalization or surgery)	179 (97, 44% / 82, 37%) (matched)	27 (9, 31% / 18, 20%)
Patients lost to follow-up (exposed/non-exposed)	0	0	32 (17/15) 7 patients with SARS-CoV-2 infection died	0
Follow-up period (exposed/non-exposed)	Median 21 (16-26) / 23 (16-25) weeks	Median 358 (32-576) / 417 days (43-581)	Mean: 7.8 ± 1.2 months	12 months
Severe COVID-19	4 (4%)	39 (16%)	NA	3 (10%)
1. Clinical worsening of IBD	•	•	•	•
2. Change of IBD therapy	-	•	-	•
3. Delay or discontinuation of IBD therapy	•	-	•	•
4. Increase in fecal calprotectin	-	-	-	-
5. Progression of UC extent	-	•	-	-
6. Change in CD location	-	•	-	-
7. Change in CD behaviour	-	•	-	-

*Exposed: patients with IBD and SARS-CoV-2 infection / Non-exposed: IBD patients without SARS-CoV-2 infection. Values represent absolute numbers (%) or medians (interquartile range). CD: Crohn’s disease. UC: ulcerative colitis. IBD: inflammatory bowel disease. NA: not available. •Outcomes evaluated in the study.

All studies matched exposed and non-exposed patients. Papa A. et al. (2022) matched them at a 1:2 ratio based on disease type (CD or UC) and biological therapy used. Hong S.J. et al. (2022) and Bezzio C. et al. (2023) matched them 1:1 exposed and non-exposed individuals based on sex, age, and diagnosis (CD or UC), and Bezzio C. et al. (2023) matched them also for therapy and disease activity. Neri B. et al (2023) enrolled four non-exposed IBD patients for each exposed IBD case. The increase in fecal calprotectin has not been evaluated by any of the four studies (Table 2).

The most frequent symptom related to SARS-CoV-2 infection was fever (72%), followed by weakness and headache (52%), and the least common symptom was dyspnea (21%). Only 12% had severe COVID-19.

Hospitalization rates due to COVID-19 among patients with IBD varied across studies. Papa A. et al. (2022) reported hospitalization in 4 of 95 patients (4.2%). Neri B. et al. (2023) observed a hospitalization rate of 10% (3 of 29 patients), with no need for intensive care unit admission. Bezzio C. et al. (2022) focused primarily on IBD flares and therapy discontinuation and did not provide data on COVID-19-related hospitalizations. In the largest cohort, Hong S.J. et al. (2022) identified severe COVID-19-defined as requiring hospitalization, oxygen supplementation, mechanical ventilation, or ICU admission-in 39 of 251 patients (16%). Taken together, these data correspond to 46 hospitalizations among 375 patients, resulting in an overall hospitalization rate of 12.3% across studies reporting this outcome, with variability depending on study design and population characteristics.

Papa A. et al. (2022) included only IBD patients receiving biologic therapy, whereas Bezzio C. et al. (2023) matched patients by therapy. Among studies that also included other IBD therapies but did not match patients by therapy^14^ ^,^ ^16^, 62% (387 of 620) received biologics, with an even higher rate in the exposed group (69%, 193 of 280). Anti-TNF agents were the most commonly used biologic therapy (463 of 1,058; 44%), with usage ranging from 27% to 78%, showing similar rates between exposed and non-exposed groups.

### Outcomes of IBD

Clinical IBD worsening and change of IBD therapy occurred with similar frequency in exposed and non-exposed. Delay or discontinuation of biological therapy occurred in half of the SARS-CoV-2 group and only in 7% of non-infected. Progression in UC extent, change in CD location, and change in CD behavior occurred in a minority of individuals, without differences between groups (Table 3).

**TABLE 3:** Outcomes of Inflammatory Bowel Disease in patients with SARS-CoV-2 infection compared without infection.

Outcomes		Papa A.	Hong S.J.	Bezzio C.	Neri B.	N / Total
		2022	2022	2023	2023	
Clinical IBD worsening	Exposed	11 (11.6%)	29 (12%)	44 (21.7%)	8 (27.5%)	92 / 578 (16%)
	Non-exposed	21 (11.3%)	38 (15%)	36 (16.7%)	19 (21%)	114 / 746 (15%)
Change of IBD therapy	Exposed	NA	94 (37%)	NA	5 (17%)	99 / 280 (35%)
	Non-exposed	NA	122 (48%)	NA	11(12.3%)	133 / 340 (40%)
Delay or discontinuation of biological therapy	Exposed	46 (48%)	63 (35%)	67 (55%)	1 (9%)	177 / 409 (43%)
	Non-exposed	7 (3.7%)	NA	15 (12%)	2 (6%)	24 / 343 (7%)
Progression of UC extent	Exposed	NA	5 (4.5%)	NA	NA	5 / 112 (4.5%)
	Non-exposed	NA	4 (3.6%)	NA	NA	4 / 112 (3.6%)
Change in CD location	Exposed	NA	1 (0.7%)	NA	NA	1 / 139 (0.7%)
	Non-exposed	NA	1(0.7%)	NA	NA	1 / 139 (0.7%)
Change in CD behavior	Exposed	NA	14 (10%)	NA	NA	14 / 139 (10%)
	Non-exposed	NA	14 (10%)	NA	NA	14 / 139 (10%)

Exposed: patients with IBD and SARS-CoV-2 infection / Non-exposed: IBD patients without SARS-CoV-2 infection. CD: Crohn’s disease. UC: ulcerative colitis. IBD: inflammatory bowel disease; NA: not available.

Two studies associated IBD flare with delay or discontinuation of IBD biological therapy. IBD flare was significantly higher in those who delayed or discontinued therapy, as can be seen in Table 4.

**TABLE 4:** Frequency of flare of Inflammatory Bowel Disease considering delay or discontinuation of biological therapy.

	IBD Flare with delayed or discontinued biological therapy	IBD Flare without delay or discontinuation of biological therapy	***P* value**
Papa A. et al., 2022	15 / 32 (46.9%)	38 / 253 (15.0%)	< .001
Bezzio C. et al., 2023	29 / 50 (58%)	53 / 190 (27.9%)	< .001
Total	44 / 82 (52%)	81 / 443 (18%)	<.0001

IBD: inflammatory bowel disease.

**FIGURE 2:**
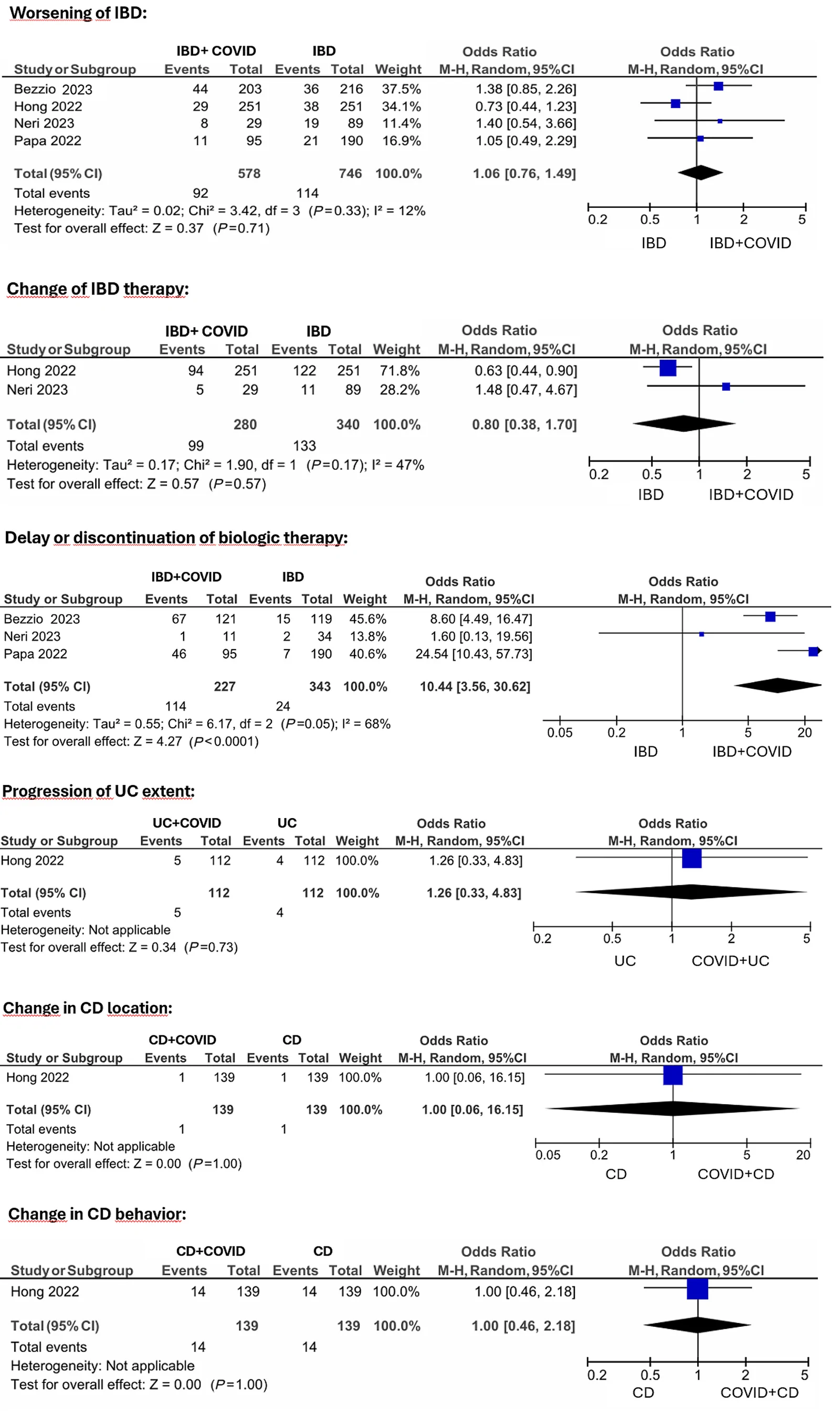
Forest plot of outcomes of inflammatory bowel disease (IBD) in patients who had SARS-CoV-2 infection compared with IBD patients without SARS-CoV-2 infection.

Our meta-analysis found that clinical worsening of IBD (OR: 1.06; 95%CI: 0.76-1.49), change of IBD therapy (OR: 0.80; 95%CI: 0.38-1.70), progression of UC extent (OR: 1.26; 95%CI: 0.33-4.83), change in CD location (OR: 1.00; 95%CI: 0.06-16.15), and change in CD behavior (OR: 1.00; 95%CI: 0.46-2.18) were not significantly different between SARS-CoV-2 infected and non-infected groups. By contrast, IBD patients who had SARS-CoV-2 infection were at higher risk of delaying or discontinuing biological therapy (OR: 10.44; 95%CI: 3.56-30.62) (Figure 2).

## DISCUSSION

This systematic review aimed to investigate outcomes in IBD following SARS-CoV-2 infection. We found only four studies comparing exposure to the infection with non-exposed IBD patients. Interestingly, all reported case-control studies, in reality, evaluated outcomes based on exposure to the infection or not; thus, they could not be considered to have a classic case-control design. Clinical worsening of IBD, change of IBD therapy, progression of UC extent, change in CD location, and change in CD behavior were not significantly different between SARS-CoV-2-infected and non-infected groups. By contrast, IBD patients who had SARS-CoV-2 infection were at higher risk of delaying or discontinuing biological therapy, and this delay/discontinuation was associated with IBD flare.

The studies evaluated were mostly Italian, with a low risk of bias, and matched exposed and non-exposed patients. The majority considered SARS-CoV-2 infection, regardless of the presence of symptoms, and only one study^14^ described the COVID-19 symptoms in IBD patients, being the main fever, weakness, and headache, similar to other studies^3^ ^,^ ^5^ ^,^ ^18^. Only 12% had severe COVID-19, but a lower rate was found in other studies, around 5-8%^5^ ^,^ ^8^ ^,^ ^18^.

Patients with IBD appear to have a higher risk of hospitalization for COVID-19 compared with the general population^19^. However, they do not seem to be at increased risk of intensive care unit admission, COVID-19-related death, or death within 30 days of diagnosis. Since our analysis included only IBD patients, we were unable to directly compare hospitalization rates with those of non-IBD individuals within the same cohorts. Nevertheless, the overall hospitalization rate observed in our review was higher than that reported in the general population. This difference may be partially explained by greater access to medical care and earlier reporting of symptoms among IBD patients, as well as by the fact that many of them are treated with immunosuppressive or biologic therapies, which could prompt physicians to pursue hospitalization more readily than in the general population.

The flare of IBD post-SARS-CoV-2 infection was low (16%) and similar to that in IBD patients without infection (15%). Some studies found a lower rate of IBD flare post-SARS-CoV-2 infection, but they did not have an IBD comparison group without infection^5^ ^,^ ^18^ ^,^ ^20^. COVID-19 and other viral infections can trigger or aggravate autoimmune diseases by activating initial innate immunity, leading to aberrations in adaptive immune cells^21^. Studies have reported the connection between enteric infection and the subsequent development of IBD^22^. Based on this, we hypothesized that SARS-CoV-2 could be a trigger for IBD exacerbation. The penetration of SARS-CoV-2 into intestinal cells results in increased TNF-α production and a heightened T-helper-17 lymphocyte response, which together induce immune dysregulation observed in patients with IBD^23^. Several autoimmune diseases involving multiple organs have occurred among patients infected with COVID-19^21^. However, our data did not corroborate this hypothesis. Understanding that SARS-CoV-2 infection does not directly worsen IBD is crucial to reduce patient anxiety and to ensure that therapeutic decisions are based on up-to-date scientific evidence, promoting effective management of IBD during SARS-CoV-2 infection.

In one of the studies included in our systematic review, all patients received biologic therapy^17^, while another study matched patients according to therapy^15^. In the remaining two studies^14^ ^,^ ^16^, 62% of patients were on biologic treatment. It is important to note that most of these studies were conducted in private health care services. Biological drugs are an expensive therapy used in moderate to severe cases of IBD, generally reserved for those who have not responded to conventional treatments. The percentage of use of biologicals in Brazil, in public services, and in reference centers for the treatment of IBD, was lower than that found in this systematic review, around 30% to 40%^3^ ^,^ ^24^. We found no data in the literature on the percentage of use of biologicals in the treatment of IBD in private health services in Brazil. A study carried out in 11 centers in Europe and another from North America, Asia, Europe, and Brazil, all public health services, showed a higher rate of use of biological therapy in IBD patients (86% and 72%)^5^ ^,^ ^8^. It should be noted that factors such as access to medicines, public health policies, and demographic characteristics influence the prevalence of biological therapies. Therefore, the percentage of IBD patients who use biological therapies can vary significantly between different studies and regions. Further studies on the prevalence of the use of biological therapy in IBD patients in Brazil are needed, both in public services and especially in private services, since data are scarce in the literature.

Regarding IBD patients who had SARS-CoV-2 infection, the rate of biologic therapy use was even slightly higher (69%). It would be reasonable to assume that patients using biologics would be at higher risk of COVID-19, as they are at higher risk of infections in general. However, this association has not been confirmed in previous studies^3^ ^,^ ^8^. There is wide variation in the literature regarding the use of biologic therapy in patients with IBD and SARS-CoV-2 infection. Wetwittayakhlang P (2021), Amiot A (2023), and Ferreira SDC (2022) had rates similar to this review. Latin American patients from the SECURE-IBD registry^25^ had 54%, and Guerra I (2021) found only 6% use of biologic therapy in patients with IBD and SARS-CoV-2 infection. We believe that this variation may be a consequence of the insecurity of the medical team at the time the studies were carried out, faced with a new and challenging scenario (COVID-19 in a patient using biologicals), which resulted in different clinical conducts.

Among patients receiving biologic therapy, we observed a predominance of anti-TNF agents (51%), consistent with findings from other studies^5^ ^,^ ^8^ ^,^ ^21^ ^,^ ^25^ ^,^ ^26^. Early in the pandemic, when data on COVID-19 outcomes were limited, some clinicians favored biologic agents perceived to have a more favorable safety profile, such as vedolizumab and ustekinumab. Nevertheless, anti-TNF therapies have remained the most widely prescribed biologics for IBD, despite the increasing availability of newer agents.

Among IBD patients who were on biologic therapy, there was a higher rate of delay or discontinuation of this therapy in the exposed group (43% vs 8%). We believe this was due to physicians’ fear of the potential adverse effects of biological therapies in infected patients. In addition, some patients discontinued biologic therapy on their own when infected. Biologic delay or discontinuation was associated with IBD exacerbation (52% vs 18%). Three of the studies included in our review were from Italy, and it has been previously reported that COVID-19 cases in Italy were associated with higher odds of discontinuation of IBD medication compared with cases in other countries^27^. Wetwittayakhlang P (2021) found a lower rate of biologic therapy discontinuation (37%) in IBD patients with active COVID-19 infection and did not find an association between biologic therapy discontinuation and IBD flare. In the SECURE-IBD data^8^, biologic therapy was discontinued in 25-40% of IBD patients due to COVID-19 infection, but the study did not assess whether IBD flare occurred in these patients.

IBD flare was not associated with SARS-CoV-2 infection but with the delay or discontinuation of biological therapy. It is already known that there is a correlation between IBD flare and lower biological drug levels^28^ ^,^ ^29^ that occur when the treatment is discontinued. 

Importantly, these findings challenge a blanket policy of routinely suspending biologics in all IBD patients with COVID-19. Rather than reflexive interruption, they support a risk-stratified approach in which biologic continuity may be considered in appropriate, lower-risk patients to prevent flares and reduce unnecessary corticosteroid or emergency-department use. This can help shift practice from virus-centric decisions to patient-specific IBD management.

Our study has limitations. Only one outcome was analyzed in all four studies, and three outcomes were evaluated in just one study each. Furthermore, definitions of SARS-CoV-2 infection and COVID-19 varied across studies.

## CONCLUSION

SARS-CoV-2 infection does not appear to alter the extent of UC, location, or behavior of CD, and is not a significant risk factor for IBD recurrence in patients undergoing biologic therapy.

The main impact appears to be on IBD treatment, especially in patients who discontinued biologic therapy, which in turn appears to be associated with IBD exacerbation.

Patients with IBD receiving biologic therapy who develop COVID-19 should be evaluated on a case-by-case basis. In the absence of risk factors for severe COVID-19, continuation of biologic therapy should be considered, given the potential risk of IBD exacerbation upon discontinuation.

## Data Availability

Data-available-upon-request
